# A CRISPR-Cas9-Mediated Large-Fragment Assembly Method for Cloning Genomes and Biosynthetic Gene Cluster

**DOI:** 10.3390/microorganisms12071462

**Published:** 2024-07-18

**Authors:** Yujing Guo, Guang Cai, Huiying Li, Zhenquan Lin, Shuobo Shi, Jin Jin, Zihe Liu

**Affiliations:** State Key Laboratory of Chemical Resource Engineering, Beijing Advanced Innovation Center for Soft Matter Science and Engineering, College of Life Science and Technology, Beijing University of Chemical Technology, Beijing 100029, Chinashishuobo@mail.buct.edu.cn (S.S.)

**Keywords:** CRISPR/Cas9, *in vitro* DNA assembly, synthetic genomes, biosynthetic gene cluster

## Abstract

The ability to clone large DNA fragments from genomes is valuable for both basic and applied research, such as the construction of synthetic genomes, and the expression of biosynthetic gene clusters (BGCs) for natural product discovery. Here, we report a fast and efficient platform for the direct capture of genome DNAs, by combining CRISPR and Gibson assembly. We demonstrate this method with the ability of cloning large DNA fragments ranging from 30 to 77 kb from various host genomes, achieving a near 100% cloning fidelity for DNA fragments below 50 kb. We next demonstrate this method by the cloning of a 40 kb fragment from *Streptomyces ceruleus* A3(2), which is rich in BGCs for natural products; and used this method cloning the 40 kb fengycin synthetic gene cluster from *B. subtilis* 168, encoding for a class of peptides with bioactivity. This method provides efficient and simple opportunities for assembling large DNA constructs from distant sources.

## 1. Introduction

Recombinant DNA construction plays a crucial role in biotechnology. While there are well-established methods for constructing short DNA fragments, the efficient construction of DNAs over 10 kb remains challenging [1,2]. As research advances, the demand for DNA engineering on a larger scale is increasing. Consequently, there is an increased demand for synthesizing and assembling extensive DNA fragments. However, the construction and expression of these large DNA fragments face challenges, which limit our ability to fully understand and manipulate biological systems [3]. Currently, significant efforts are underway to synthesize DNA for various genomes, including Mycoplasma mycoides, recoded *Escherichia coli*, and synthetic *Saccharomyces cerevisiae* (Sc2.0). Furthermore, ongoing projects involve the synthesis of artificial genomes for other species, such as cyanophages. In contrast, natural products (NPs) serve as major sources for bio-antibiotic, pharmaceutical, and biofuel production. Most NPs are produced by biosynthetic gene clusters (BGCs) [4,5,6]. As high-throughput sequencing and bioinformatics continue to advance rapidly [7,8], heterologous expression has emerged as a crucial approach for exploring novel natural products [9]. However, these gene clusters typically span tens to hundreds of kilobases, posing challenges for cloning [10]. To facilitate gene expression, large DNA fragments must be cloned. However, current methods have limitations, and there is a need to develop a simpler and more convenient method with fewer restrictions [2,11,12]. Hence, there is an urgent need to develop efficient and user-friendly tools for cloning large-scale DNA fragments.

A variety of methods have been reported to directly clone these BGCs [13,14,15]. In Table 1, we briefly list some of the most used methods of cloning large-fragment DNA. For example, transformation-associated recombination (TAR), a cloning method based on the efficient homologous recombination ability of yeast [16,17,18], makes it challenging to extract plasmids from yeast, thereby complicating restriction analysis. Exonuclease combined with RecET recombination (ExoCET), which integrates *in vitro* annealing and in vivo recombination of *E. coli*, addresses the challenge of challenging identification and plasmid acquisition. However, biosynthetic gene clusters (BGCs) are acquired through enzyme digestion [19,20]. The restriction sites’ limitations may hinder the release of gene clusters [12,21]. In addition, Cas9-assisted targeting of chromosome segments (CATCH) [22,23] and the CRISPR/Cas12a-mediated fast direct biosynthetic gene cluster cloning platform (CAT-FASHING) [24] are not restricted by restriction sites. However, these methods utilize the agarose gel embedding technique to extract the genome and BGCs, which significantly increases the complexity of the operation [25]. There are also examples of applying Cre/loxP systems [26,27], phage integrase-mediated site-specific recombination [28], the λ packaging system for cloning [14], and the TelN/tos system [29], but these approaches are time consuming. In addition, these experiments are difficult to operate.

In this study, we have successfully developed a method that combines the simplicity and accuracy required for cloning large DNA fragments. By leveraging the CRISPR/Cas9 system, we have developed an *in vitro* system for the targeted recognition and cleavage of genomic DNA. This, coupled with Gibson cloning technology, enables the seamless connection of large DNA fragments and vectors. As a result, we achieved rapid and precise construction of macromolecular recombinant plasmids, with a maximum clone length of 77 kb. We selected the most cited and classical methods to compare with our own. Our method surpasses other existing techniques for large-fragment DNA cloning in terms of its simplicity, ease of use, high fidelity, and shorter turnaround time.

## 2. Materials and Method

### 2.1. Synthesis and Purification of sgRNA

The design resources from the Zhang lab (https://www.zlab.bio/Resources-guidedesign, accessed on 9 July 2024) were used to create 20 bp oligonucleotides.

The double-stranded transcription template DNA was obtained by PCR annealing; the primers are shown in Table S1. The sgRNA-E-F and sgRNA-scaffold-R primers were used to generate the DNA template for sgRNA. The 15 kb DNA fragment was cloned using sgRNA E1 and sgRNA E2. The 30 kb DNA fragment was cloned using sgRNA E1 and sgRNA E3. The 50 kb DNA fragment was cloned using sgRNA E1 and sgRNA E4. The 60 kb DNA fragment was cloned using sgRNA E1 and sgRNA E5. The 77 kb DNA fragment was cloned using sgRNA E1 and sgRNA E6. The 100 kb DNA fragment was cloned using sgRNA E1 and sgRNA E7.

Using primers sg-sc40k-F and sgRNA-scaffold-R to obtain sgRNAsc40k, the 40 kb DNA fragment from *Streptomyces ceruleus* A3(2) was cloned using primers sgsc40k 1 and sgsc40k 2. Using primers sg-bs40k-F and sgRNA-scaffold-R to obtain sgRNAbs40k, the 40 kb DNA fragment from *B. subtilis* 168 was cloned using primers sgbs40k 1 and sgbs40k 2.

*In vitro* transcription was performed using a T7 High Yield RNA Transcription Kit (Vazyme, Nanjing, China). After transcription, large quantities of RNA were purified using VAHTS RNA Clean Beads (Vazyme, Nanjing, China). The transcription and purification methods were carried out according to the instructions.

### 2.2. Expression and Purification of Cas9

Cas9 gene fragments carrying *Sal*I and *Nco*I (Takara, Tokyo, Japan) restriction sites at the ends were obtained by PCR using primers Cas9-SalI-F and Cas9-NcoI-R. The template was a lab-preserved plasmid containing Cas9. The pET28a vector and Cas9 gene fragment were digested with *Sal*I and *Nco*I. Subsequently, the vector was ligated with the fragment by T4 DNA ligase (NEB, Ipswich, MA, USA). The primers are listed in Table S3.

The constructed plasmid was transformed into *E. coli* BL21(DE3). Then, 10 mL of seed solution was added to 1 L of fresh LB and shaken at 37 °C. When the OD_600_ reached 0.6–0.8, 1 M IPTG solution was added to achieve a final concentration of 0.4 mM. The protein expression was induced at 16 °C for 20 h. The recombinant protein was purified using AKTA (GE Healthcare, Chicago, IL, USA).

### 2.3. Preparation of Genomic DNA

*E. coli* and *B. subtilis* were cultured on LB for 12 h at 30 °C, 200 rpm, while *Streptomyces ceruleus* A3(2) was cultured on YPD for 2 days at 30 °C, 200 rpm. After centrifugation and washing with 30 mL of H_2_O, the sample was resuspended in 8 mL of SET buffer (75 mM NaCl, 25 mM ethylenediaminetetraacetic acid (EDTA), 20 mM Tris, pH 8.0) and 10 mg of lysozyme was added. After incubation at 37 °C for 1 h, 10 mg of proteinase K and 1 mL of 10% SDS were added. The mixture was then incubated at 50 °C for 2 h until the solution became clear. Gram-negative bacteria do not require lysozyme lysis for 1 h. Then, 3.5 mL of 5 M NaCl was added to the lysate. Genomic DNA was recovered from the lysate through phenol–chloroform–isoamyl alcohol (25:24:1, pH 8.0) extraction. 15 mL of phenol–chloroform–isoamyl alcohol was added, and then, it was centrifuged at 8000 rpm for 20 min. 500 μL of the supernatant was absorbed into a 2 mL centrifuge tube with a cut tip, 35 μL of 3 M sodium acetate was added, and turned upside down and mixed well. Then, 1.2 mL of anhydrous ethanol was added, mixed upside down until a white flocculent precipitated, and then stirring precipitation into a 1.5 mL centrifuge tube containing 1 mL of 75% ethanol. Centrifuged at 10,000 rpm for 2 min; the supernatant was drained and air-dried, and 100 μL of ddH_2_O was added.

### 2.4. In Vitro Cas9 Endonuclease Cleavage for Genomic DNA

Initially, 800 nM Cas9, 400 nM sgRNA1, 400 nM sgRNA2, 30 μL of 10 × NEB buffer 3.1, and 1 μL of RRI (recombinant ribonuclease inhibitor) formed a 300 μL complex at 37 °C for 20 min without the addition of genomic DNA, after which 0.02~0.04 nM DNA was introduced. It was then cultured at 37 °C for 2 h.

### 2.5. DNA Purification after In Vitro Cas9 Digestion

Adding 300 μL of equal-volume phenol–chloroform–isoamyl alcohol (25:24:1, pH 8.0) to the *in vitro* Cas9 endonuclease cleavage system, the supernatant was collected by centrifugation; and adding 20 μL of 3 M sodium acetate in the 200 μL supernatant, it was mixed well. Then, 500 μL of anhydrous ethanol was added. It was centrifuged at 10,000 rpm for 2 min. The supernatant was removed and 1 mL of 75% ethanol was added. It was centrifuged at 10,000 rpm for 2 min and the supernatant was drained, then 8 μL of ddH_2_O was added and suspended.

### 2.6. Preparation of Vector with the Homologous Arm

The vectors of large fragments cloned in this paper are pYESZ5 (Amp, p15A ori, CEN/ARS, LEU2) and pYESZ7 (Amp, p15A ori, CEN/ARS, LEU2, TEF1p, tCYC1), which are stored in the laboratory. Vectors with homologous arms were obtained by PCR; the primers are shown in Table S2.

The homologous arms were designed on primers, using vector pYESZ5 as a template (*E. coli* and yeast shuttle plasmid); 2 × Phanta Flash Master Mix Dye Plus (Vazyme, Nanjing, China) was used to obtain DNA fragments with homologous arms by PCR amplification.

### 2.7. Gibson Assembly

In the 20 μL system, 10 μL of 2 × NEBuilder Mix, 2 μL of carrier, and 8 μL of DNA purification product were used; it was then incubated for 2 h at 50 °C.

### 2.8. Dialysis

Masses of 1 g of agarose and 2 g of glucose were heated and dissolved in 100 mL of ddH_2_O. A volume of 1 mL of the mixture was taken and added to a 1.5 mL centrifuge tube, and this was put on another 1.5 mL centrifuge tube and the tip of the centrifuge tube was used to form a groove. The reaction solution was added to the groove at 4 °C for 40 min. After dialysis, the solution underwent electroporation into *E. coli* DH10B or DH5α.

### 2.9. Electroporation

A volume of 1 mL of seed solution was cultured in 100 mL of fresh LB medium at 30 °C and 220 rpm until reaching an OD_600_ of 0.6~0.8. 5000 rpm for 10 min and collected the cells. And then the cells were washed twice with 30 mL of pre-cooled ddH_2_O. Finally, they were gently resuspended in 1 mL of pre-cooled 10% glycerin. Each tube was packed with 100 μL of competent cells.

## 3. Results

### 3.1. Establishment of CRISPR/Cas9 System for Cloning Large DNA Fragments In Vitro

To address the limitations of previously reported methods, our goal is to develop a highly versatile and user-friendly cloning method. One of the critical aspects of cloning large fragments is to accurately obtain the target DNA fragments. To overcome this challenge, we have successfully established a high-fidelity *in vitro* cleavage system that allows us to obtain the desired DNA fragments with precision. This breakthrough significantly contributes to the development of our method, making it more accessible and efficient for cloning large DNA fragments.

**Figure 1:**
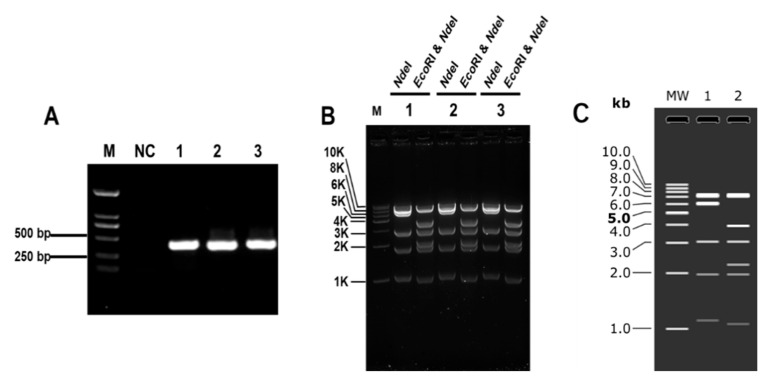
The plasmid with 15 kb DNA fragment restriction verification. (**A**) Colony PCR verification results of three blue clones on the plate. NC: negative control; M: 2000 bp Marker. (**B**) The 15 kb plasmid digestion verification result. (**C**) Simulation of agarose gel; MW: 1 kb DNA Ladder; 1: *Nde*I restriction; 2: *EcoR*I and *NdeI* restriction.

Firstly, we designed *in vitro* digestion experiments to verify the enzyme activity and efficiency. Lee et al. used a molar concentration ratio of Cas9:gRNA:genome of 100:100:1 for *in vitro* cleavage employing the CRISPR/Cas9 system [30]. In order to obtain a sufficient number of target fragments, we initially used the molar ratio of Cas9:sgRNA:DNA = 1000:1000:1. The initial amount of DNA in the cleavage system was 20–40 μg. We designed a 15 kb DNA fragment containing the *LacZ* gene of *E. coli* as the cloning marker. The cloning efficiency was verified by blue–white spot screening. As shown in Figure 1, the CRISPR/Cas9 method for cloning large DNA fragments *in vitro* was preliminarily established. The blue and white clones in the three parallel groups were counted, and the fidelity was calculated by dividing the number of blue clones by the total number of clones. The numbers of white clones on the plate were 57, 43, and 38, respectively. Only one plate obtained three blue clones, with an average fidelity of 1.7%. First, the three blue clones were verified by colony PCR. The results showed that DNA fragments were successfully connected, and the plasmids of the three clones were extracted and verified by restriction digestion. Enzyme digestion was verified by *Nde*I (7520 bp, 6215 bp, 3070 bp, 1974 bp, 1119 bp) and *EcoR*I and *NdeI* (7520 bp, 3978 bp, 3070 bp, 2237 bp, 1974 bp, 1070 bp, 49 bp). As shown in Figure 1, the results with agarose gel were consistent with the simulated results, which proved successful construction.

We successfully cloned 15 kb fragments from *E. coli*, but the efficiency and fidelity were low, and system optimization was needed for improvement.

### 3.2. Optimization of CRISPR/Cas9 System for Cloning Large DNA Fragments In Vitro

While the current system can clone 15 kb fragments from Gram-negative bacteria, it faces challenges related to low efficiency and struggles to meet higher experimental demands. To address these limitations, further optimization is necessary to increase the number of positive clones obtained. The key step in this method involves the efficient and specific cleavage of genomic DNA by Cas9-gRNA to release the target DNA. The molar ratio of Cas9, sgRNA, and DNA in the digestion system plays a crucial role in ensuring the efficient production of a sufficient amount of target DNA. Fine-tuning this ratio is essential for enhancing the overall success rate of the cloning process [22,23]. Research has shown that an excessive concentration of Cas9 protein can lead to non-specific cleavage, while a relatively low concentration can result in incomplete digestion of the target sequence, leading to a reduced yield of free targeted DNA. In conventional CRISPR/Cas9 *in vitro* experiments, the standard molar ratio between Cas9 protein and sgRNA is 1:1. While a high concentration of sgRNA is typically well tolerated, a low concentration of sgRNA may result in incomplete digestion, negatively impacting subsequent cloning procedures. Therefore, it is critical to optimize the concentrations of the Cas9 protein and sgRNA to achieve efficient and specific cleavage of genomic DNA, which is essential for successful cloning of large DNA fragments [22,23]. In addition, the ratio of carrier to fragment, and the length of homologous arm required for recombination are important factors.

**Figure 2:**
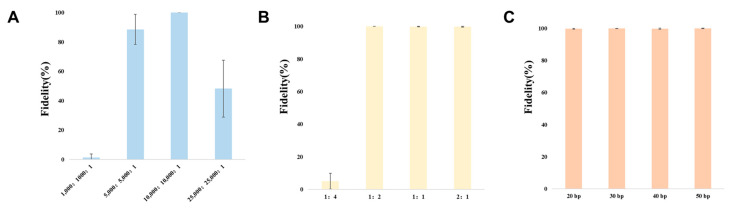
The fidelity of optimal cloning conditions. (**A**) The fidelity of the ideal molar concentration ratio (nM) for CRISPR/Cas9 cleavage. (**B**) The fidelity of the vector-to-insert fragment ratio (nM). (**C**) The fidelity of the different lengths of the homologous arm.

Based on our investigations of the *in vitro* digestion system, the vector-to-insert fragment ratio, and the length of the homologous arm, we have identified the optimal cloning conditions. As shown in Figure 2, the optimal molar concentration ratio (nM) for CRISPR/Cas9 cleavage *in vitro* is between 5000:5000:1 and 10,000:10,000:1. Under these conditions, the cloning fidelity for 15 kb DNA fragments ranges from 80% to 100%. When the ratio of vector-to-insert fragment falls within the range of 1:2 to 2:1 (as indicated in Table S4), it results in a cloning fidelity of approximately 100% for the 15 kb DNA fragment. Moreover, the cloning fidelity remains close to 100% for the 15 kb DNA fragment when using a vector with homologous arms ranging from 20 to 50 bp (as illustrated in Table S5). Interestingly, different lengths of homologous arms have little effect on cloning fidelity. This is possibly because homologous arm length is not the key influencing factor when recombinant enzyme fidelity is high enough.

Consequently, based on this data, we recommend using an optimal molar concentration ratio of 10,000:10,000:1 for sgRNA:Cas9:genome, a carrier-to-fragment ratio of 1:1, and homologous arms with lengths of 20–50 bp for cloning large DNA fragments.

### 3.3. Clones of Large DNA Fragments of Different Lengths

**Figure 3:**
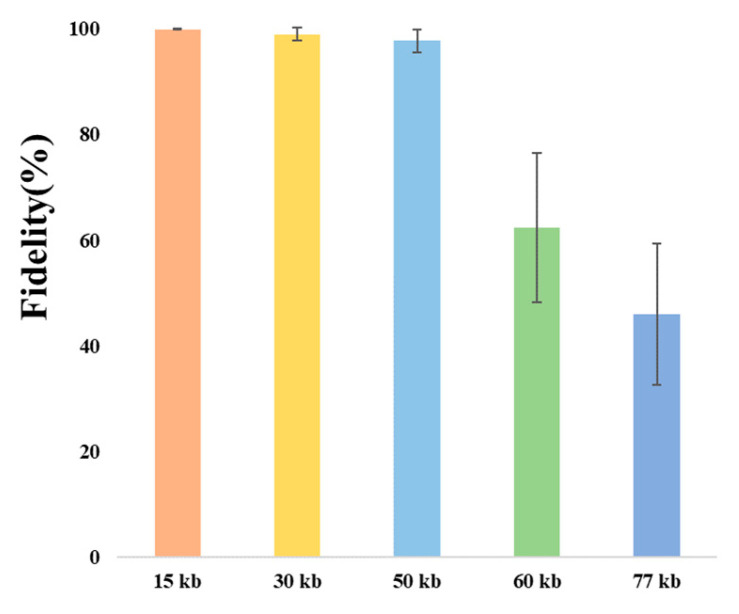
The fidelity of cloning different-length DNA fragments.

However, when assembling genomes and cloning gene clusters for natural product biosynthesis, the target DNA fragment is typically in the range of tens of kilobases. Consequently, we will investigate the maximum length of DNA that can be cloned using this method. A schematic for different-length DNA fragments for cloning is shown in Figure S1. Based on the data presented in Figure 3 and Table 2, it is evident that the cloning fidelity of DNA fragments less than 50 kb remains consistently high, ranging from 95% to 100%. When the DNA fragment exceeds 50 kb, the cloning fidelity significantly decreases. For instance, the average cloning fidelity of 60 kb DNA fragments was 62.4%, while for 77 kb DNA fragments, the average cloning fidelity dropped further to 46.03%. When the DNA fragment exceeds 77 kb, it becomes impossible to obtain correct clones using this method. The verification results for 30 kb, 50 kb, 60 kb, and 77 kb DNA fragments are shown in Figures S2–S5, respectively.

Additionally, we observed a significant difference in the number of clones obtained from clones with varying lengths of DNA fragments. As the DNA length increased, the number of blue clones sharply decreased, while the number of white clones increased. This is feasible due to the increased susceptibility of longer DNA fragments to random breaks, thereby complicating the cloning process. Although this method has limitations when cloning larger DNA fragments, it still offers advantages such as high fidelity, short cycles, and simplicity in cloning DNA fragments within the range of 30–80 kb when compared to other conventional methods.

### 3.4. Cloning Biosynthetic Gene Cluster

**Figure 4:**
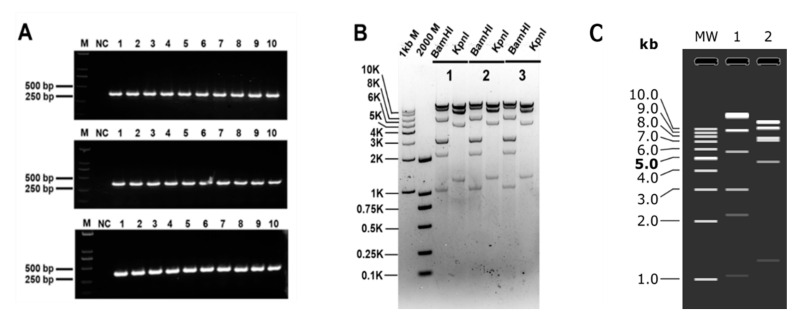
The plasmid with 40 kb DNA fragment from *Streptomyces ceruleus* A3(2) restriction verification. (**A**) Colony PCR verification; 10 clones were randomly screened in three independent replicate cloning experiments for PCR verification. M: 2000 bp DNA Marker. NC: Negative control. (**B**) *Streptomyces ceruleus* A3(2) 40 kb plasmid digestion verification results. M: 1 kb DNA Ladder; 2000 bp DNA Marker. (**C**) Simulation of agarose gel. MW: 1 kb DNA Ladder; 1: *BamH*I restriction; 2: *Kpn*I restriction.

Furthermore, we aim to broaden the application of this technique by attempting to use it for cloning DNA fragments from diverse sources. *Streptomyces ceruleus* A3(2) has a high GC content and larger genome. It is great to see that this method was successfully applied in *Streptomyces ceruleus* A3(2). This method successfully cloned the 40 kb genome of *Streptomyces ceruleus* A3(2) with an average cloning fidelity of 93%. It is worth mentioning that although the number of clones obtained on each plate is small, the clones are almost always correct (as shown in Table 3). This further demonstrates the potential of this method in cloning large DNA fragments from diverse sources. First, the ten blue clones were verified by colony PCR. The results showed that DNA fragments were successfully connected, and the plasmids of three clones out of ten were extracted and verified by restriction digestion. Enzyme digestion was verified by *BamH*I (20,204 bp, 9623 bp, 5703 bp, 3024 bp, 3013 bp, 2163 bp, 1043 bp, 33 bp) and *Kpn*I (13,144 bp, 10,640 bp, 7804 bp, 7304 bp, 4666 bp, 1248 bp). The plasmid digestion verification results are presented in Figure 4.

**Figure 5:**
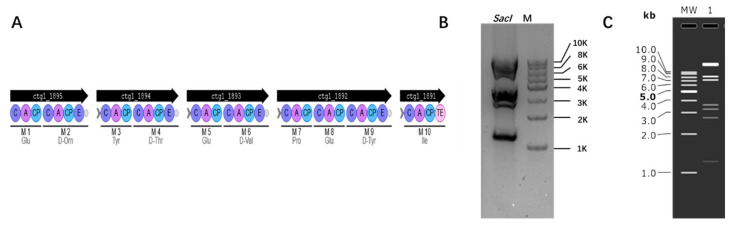
The plasmid with 40 kb DNA fragment from *B. subtilis* 168 restriction verification. (**A**) Fengycin BGC information from *B. subtilis* 168. ctg1: gene number; C: condensation domain; A: adenylation domain; CP: peptidyl carrier protein; E: epimerization domain; TE: thioesterase domains; M: module. (**B**) Plasmid digestion verification results. M: 1 kb DNA Ladder. (**C**) Simulation of agarose gel. MW: 1 kb DNA Ladder; 1: *Sac*I restriction.

In addition, due to the necessity for natural product extraction, we delved into the potential of cloning biosynthetic gene clusters using this method. *Bacillus*, a strain capable of producing various secondary metabolites and widely utilized in medicine and the chemical industry, was chosen as our model organism. The biosynthetic gene cluster information of *Bacillus* was obtained through antiSMASH. Based on the information provided by the prediction, we selected fengycin as the target. Fengycin has good biological activity and acts as an antibacterial agent. We successfully cloned a 40 kb fengycin cluster from *Bacillus subtilis* 168 [31], and the BGC information is shown in Figure 5A. First, the clones were verified by colony PCR, and the plasmids of the correct clones were extracted and verified by restriction digestion. The results of the plasmid digestion verification are presented in Figure 5B. Enzyme digestion was verified by *Sac*I (16,751 bp, 8260 bp, 7167 bp, 3582 bp, 3276 bp, 2706 bp, 1218 bp).

## 4. Discussion and Conclusions

Herein, we describe a method for cloning large fragments of DNA, which can be used to reconstruct the genome, as well as to clone biosynthetic gene clusters. Compared with previous methods, our method is more efficient, easier, has short cycles, and is capable of cloning genomes of different species. Although the length of the fragment we cloned seems to be short, it can meet the general cloning needs. While this approach does not show fidelity for cloning DNA fragments larger than 80 k, most biosynthetic gene clusters are less than 80 k.

The fidelity of cloning large DNA is indeed positively correlated with the quality of the genomic DNA [29,32]. Therefore, ensuring a high-molecular-weight DNA genome is indeed a key factor for the successful cloning of large DNA fragments [24]. In this study, genomic DNA was extracted using the phenol–chloroform method, which was found to be simpler and faster than the reported embedding method [15]. By using CRISPR/Cas9 to cleave genomic DNA and obtain target DNA fragments, we significantly reduced the time and cost of target screening [15]. We found that the fidelity of cloning DNA fragments smaller than 50 kb using this method was nearly 100%. When attempting to clone 77 kb of DNA, the average fidelity dropped to 46%. Moreover, as the cloned DNA fragments increased in size, the number of clones on the plate decreased significantly, and partial false positives emerged during blue–white screening. This could be due to the damage caused to the genome during extraction [24]. We have further extended the universality of this method, which can be applied not only to artificial genome construction but also to cloning large fragments of DNA from various sources, including BGCs without restriction. This method successfully cloned a 40 kb large fragment of DNA from *Streptomyces ceruleus* A3(2), with an average cloning fidelity of 93%, and a 40 kb BGC from Bacillus subtilis 168. This provides a new approach for directly cloning gene clusters without the requirement for restriction sites. In conclusion, this study introduces a straightforward and effective method for cloning large DNA fragments, which can be broadly utilized for gene clusters or large DNA fragments from various strains.

## Figures and Tables

**Table 1:** Comparison of different methods for large DNA cloning [15].

	Advantages	Disadvantages	DNA Fragments	Fidelity	Cycle
LLHR [21]	Technically easier; suitable for cloning small- and mid-BGCs; simple to use for recombination with short homologous arms.	False positives; difficult to clone large-size BGCs.	<~52 kb	<~50%	~3 d
ExoCET [19]	Technically easier; simple to use for recombination with short homologous arms.	Low efficiency for cloning large-size BGCs.	<~106 kb	4~100%	~3 d
TAR-CRISPR [18]	Cas9-facilitated high-efficiency cloning; suitable for cloning large genomic regions.	Technically challenging to use yeast spheroplasts for highly efficient transformation; some false positives; requires careful preparation and/or manipulation of gDNA.	-	<35%	~7 d
CATCH [22]	Suitable for cloning large genomic regions.	Requires careful preparation of the genomic DNA in gel.	<~150 kb	2~90%	~4 d
CAT-FISHING [24]	Suitable for cloning large genomic regions with high GC.	Low efficiency.	<~145 kb	8~55%	3~4 d
This method	Technically easier; short cycle; high fidelity; can clone large fragments from different sources.		<~80 kb	46–100%	~2.5 d

**Table 2:** The colony count and efficiency of cloning different-length DNA fragments.

	Group	Efficiency	Fidelity	Average Fidelity
15 kb	1	448/1	99.8%	99.9%
	2	628/0	100%	
	3	366/0	100%	
30 kb	1	124/3	97.6%	99.0%
	2	79/0	100%	
	3	179/1	99.4%	
50 kb	1	46/0	100%	97.7%
	2	37/1	97.4%	
	3	67/3	95.7%	
60 kb	1	12/14	46.1%	62.4%
	2	18/8	69.2%	
	3	23/9	71.9%	
77 kb	1	9/6	60%	46.0%
	2	15/14	44.8%	
	3	6/12	33.3%	
100 kb	1	0/0	0	N/A
	2	0/12	0	
	3	0/6	0	

**Table 3:** The efficiency and fidelity of cloning *Streptomyces* 40 kb DNA fragment.

Group	Efficiency	Fidelity	Average Fidelity
**1**	27/30	90%	93%
**2**	39/42	92.8%	
**3**	19/20	95%	

## Data Availability

The original contributions presented in the study are included in the article/Supplementary Material, further inquiries can be directed to the corresponding authors.

## Supplementary Materials

The following supporting information can be downloaded at: https://www.mdpi.com/article/10.3390/microorganisms12071462/s1, Figure S1: The scheme of design different length DNA fragments for cloning; Figure S2: The plasmid with 30 kb DNA fragment restriction verification; Figure S3: The plasmid with 50 kb DNA fragment restriction verification; Figure S4: The plasmid with 60 kb DNA fragment restriction verification; Figure S5: The plasmid with 77 kb DNA fragment restriction verification.; Table S1: sgRNA transcription template preparation primers in vitro; Table S2: Large DNA fragment clone homology arm vector backbone amplification primers; Table S3: Other primers used in this study; Table S4: Assembly system optimization; Table S5: The homologous arm length optimization.
