# Supplementary material for: A CRISPR-Cas9-Mediated Large-Fragment Assembly Method for Cloning Genomes and Biosynthetic Gene Cluster
Source: Microorganisms. 2024 Jul 18;12(7):1462. doi: 10.3390/microorganisms12071462 (PMC11279360; doi:10.3390/microorganisms12071462)
Supplement: Supplementary file 1 [file microorganisms-12-01462-s001.zip › microorganisms-3098933-supplementary.pdf]

Supporting information

A CRISPR-Cas9-Mediated Large-Fragment Assembly Method for Cloning Genomes and Biosynthetic Gene Cluster

Yujing Guo, Guang Cai, Huiying Li, Zhenquan Lin, Shuobo Shi, Jin Jin * and Zihe Liu *

State Key Laboratory of Chemical Resource Engineering, Beijing Advanced Innovation Center for Soft Matter Science and Engineering, College of Life Science and Technology, Beijing University of Chemical Technology, Beijing 100029, China; 2021400337@buct.edu.cn (Y.G.); guangcai_july@163.com (G.C.); 17861120057@163.com (H.L.); linzq2@126.com (Z.L.); shishuobo@mail.buct.edu.cn (S.S.)

* Correspondence: 2015207264@tju.edu.cn (J.J.); zihe@mail.buct.edu.cn (Z.L.)

Supplementary Table S1 sgRNA transcription template preparation primers *in vitro*

Name	Sequence
sgRNA-scaffold-R	AAAAAAAAGCACCGACTCGGTGCCACTTTTTCAAGTTGATAACGGACTAGCCTTATTTTAACTTGCTATTTCTAGCTCTAAAAC
sgRNA-E1-F	GGATTCTAATACGACTCACTATAGGGgaacaattcccggtaaccagGTTTTAGAGCTAGAAATAGC
sgRNA-E2-F	GGATTCTAATACGACTCACTATAGGGacgaatcctgaaaagcgtggGTTTTAGAGCTAGAAATAGC
sgRNA-E3-F	GGATTCTAATACGACTCACTATAGGGggtgatacgctctggaccagGTTTTAGAGCTAGAAATAGC
sgRNA-E4-F	GGATTCTAATACGACTCACTATAGGGgtacgaataaatcgcgatggGTTTTAGAGCTAGAAATAGC
sgRNA-E5-F	GGATTCTAATACGACTCACTATAGGGtgtgaaacgttagagagacgGTTTTAGAGCTAGAAATAGC
sgRNA-E6-F	GGATTCTAATACGACTCACTATAGGGacaccagacactgccgacggGTTTTAGAGCTAGAAATAGC
sgRNA-E7-F	GGATTCTAATACGACTCACTATAGGGcgataacgtaatcgtccaggGTTTTAGAGCTAGAAATAGC
sg-sc40k-1F	GGATTCTAATACGACTCACTATAGGGtcgtctgccagtaggttccgGTTTTAGAGCTAGAAATAGC
sg-sc40k-2F	GGATTCTAATACGACTCACTATAGGGgacactctcgaagttgcatgGTTTTAGAGCTAGAAATAGC
sg-bs40k-1F	GGATTCTAATACGACTCACTATAGGGagcgtgccggcttctaacttGTTTTAGAGCTAGAAATAGC
sg-bs40k-2F	GGATTCTAATACGACTCACTATAGGGgttccgcccaccattctgtcGTTTTAGAGCTAGAAATAGC

Supplementary Table S2 Large DNA fragment clone homology arm vector backbone amplification primers

Name	Sequence	Plasmid
PY-E15k-HA-R	accgcgttgaccggaacaattcccggtaacAAGTAATTGGTTGTTTGGCC	15 kb DNA fragment from *E. coli* and optimization of homology arm length (30 bp)
PY-E15K-30-HA-F	cggatcttaatgcacgaatcctgaaaagcgTCTTCCGCTTCCTTTTACTC	
PY-E15K-20-HA-F	tgcacgaatcctgaaaagcgTCTTCCGCTTCCTTTTACTC	optimization of homology arm length (20 bp)
PY-E15k-20-HA-R	ccggaacaattcccggtaacAAGTAATTGGTTGTTTGGCC	
PY-E15K-40-HA-F	ggttcgcgatcggatcttaatgcacgaatcctgaaaagcgTCTTCCGCTTCCTTTTACTC	optimization of homology arm length (40 bp)
PY-E15k-40-HA-R	gccacctaataccgcgttgaccggaacaattcccggtaacAAGTAATTGGTTGTTTGGCC	
PY-E15K-50-HA-F	cgttaatagtggttcgcgatcggatcttaatgcacgaatcctgaaaagcgTCTTCCGCTTCCTTTTACTC	optimization of homology arm length (50 bp)
PY-E15k-50-HA-R	agctcagcgcgccacctaataccgcgttgaccggaacaattcccggtaacAAGTAATTGGTTGTTTGGCC	
PY-E30K-HA-F	accttcgaccggcggtgatacgctctggacTCTTCCGCTTCCTTTTACTC	30 kb DNA fragment from *E. coli*
PY-E50k-HA-F	gtagcaaaccagagtttacgcgtgccgccaTCTTCCGCTTCCTTTTACTC	50 kb DNA fragment from *E. coli*
PY-E60k-HA-F	attttgcgattgtgaaacgttagagagacgTCTTCCGCTTCCTTTTACTC	60 kb DNA fragment from *E. coli*
PY-E77k-HA-F	cgtgggcgagggttcatccgtacgccgccgTCTTCCGCTTCCTTTTACTC	77 kb DNA fragment from *E. coli*
PY-E100k-HA-F	accgacgccgcatgaaattcgcaaccacctTCTTCCGCTTCCTTTTACTC	100 kb DNA fragment from *E. coli*
PY-sc40k-HA-R	cgtcggcaaggcctacgacggcgaccccggAAGTAATTGGTTGTTTGGCC	40 kb DNA fragment from *Streptomyces ceruleus* A3(2)
PY-sc40k-HA-F	cgtgtggacctgtgtccgtagtgtccccatTCTTCCGCTTCCTTTTACTC	
PY-bs40k-HA-F	aaagaaaaggattgttgatcaaaacgggcatgtagatgaaaaaagcgtgccggcttctaagcgctaGCGGAGTGTATACTGGCTT	40 kb DNA fragment from *B. subtilis* 168
PY-bs40k-HA-R	tacttttccttgtgcaggagtgcctttcatgcggtttctcagcgttccgcccaccattctAACTGTGGGAATACTCAGGTATCGT	

Supplementary Table S3 Other primers used in this study

Name	Sequence	
Cas9-SalI-F	GCTCCGTCGACTTAGTGATGGTGGTGGTGATGGGATCCGCCGTCGTCACCTCCTAGCTGACTCAAATC	pET28a-Cas9
Cas9-NcoI-R	AACAACGGTCTCCCATGGATAAGAAATACTCAATAG	
Cas9-V-F	CTCAGCGGTGGCAGCAG	pET28a-Cas9 Colony PCR verification
Cas9-V-R	GTGATGCCGGCCACGATGC	
P-V-F	CTTACGATACCTGAGTATTCCCACAG	DNA fragment from *E. coli* colony PCR verification
E-V-R	TGAATATGTGGCTCCACAAACGG	
Sc-V-R	GTGTCGGCCCTGTTGATCGCC	DNA fragment from *S. ceruleus* A3(2) colony PCR verification

Supplementary Table S4 Assembly system optimization

insert：vector	Group	Efficiency	Fidelity	Average Fidelity
1：4	1	0/16	0	5%
	2	2/36	5.3%	
	3	3/28	9.7%	
1：2	1	86/0	100%	100%
	2	317/0	100%	
	3	189/0	100%	
1：1	1	448/1	99.7%	99.9%
	2	628/0	100%	
	3	366/0	100%	
2：1	1	156/0	100%	99.8%
	2	219/1	99.5%	
	3	98/0	100%	

Supplementary Table S5 The homologous arm length optimization

	Group	Efficiency	Fidelity	Average Fidelity
	1	298/1	99.7%	
20 bp	2	378/0	100%	99.7%
	3	498/2	99.6%	
	1	448/1	99.8%	
30 bp	2	628/0	100%	99.9%
	3	366/0	100%	
	1	571/0	100%	
40 bp	2	513/0	100%	99.8%
	3	389/2	99.5%	
	1	519/0	100%	
50 bp	2	418/1	99.7%	99.9%
	3	698/0	100%	

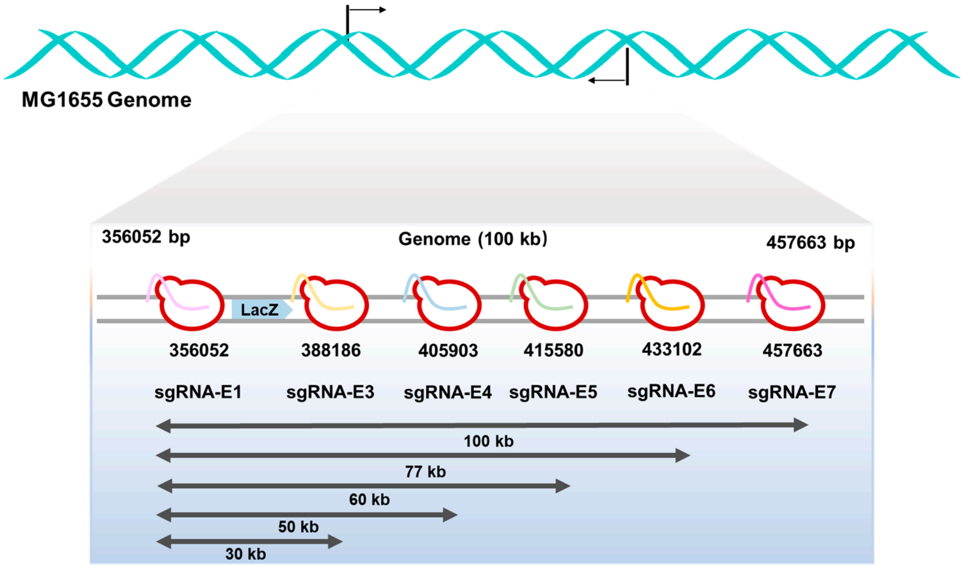

Supplementary Figure S1 The scheme of design different length DNA fragments for cloning

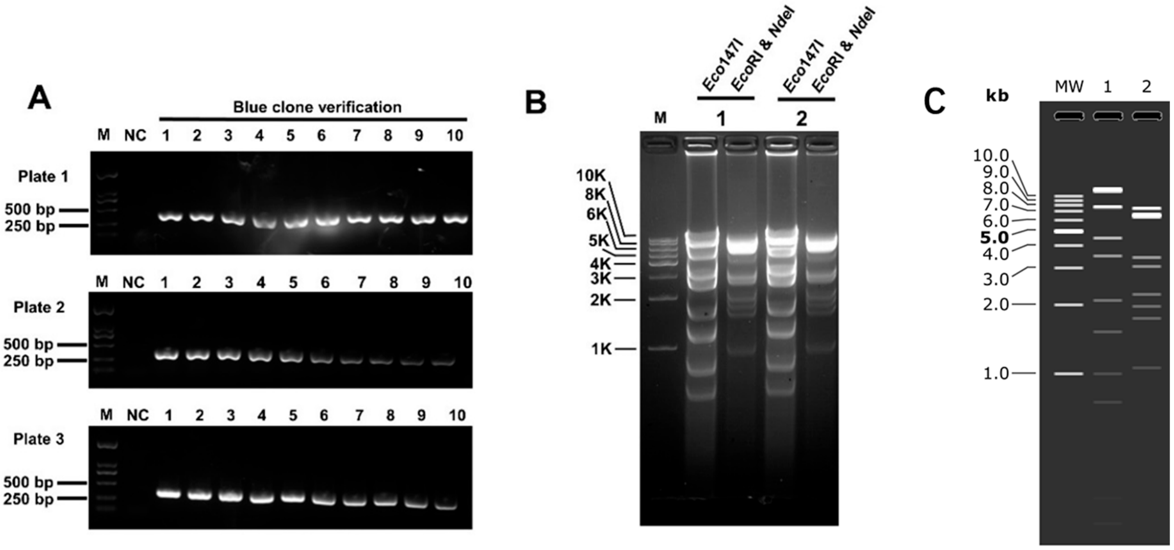

Supplementary Figure S2 The plasmid with 30 kb DNA fragment restriction verification.

(A) Colony PCR verification, 10 transformants were randomly screened in three independent replicate cloning experiments for PCR verification. (B) *E. coli* 30 kb plasmid digestion verification results. M: Maker. (C) Simulate agarose gel. MW: 1 kb DNA Ladder; 1: *Eco147*I; 2: *Eco*RI &*Nde*I.

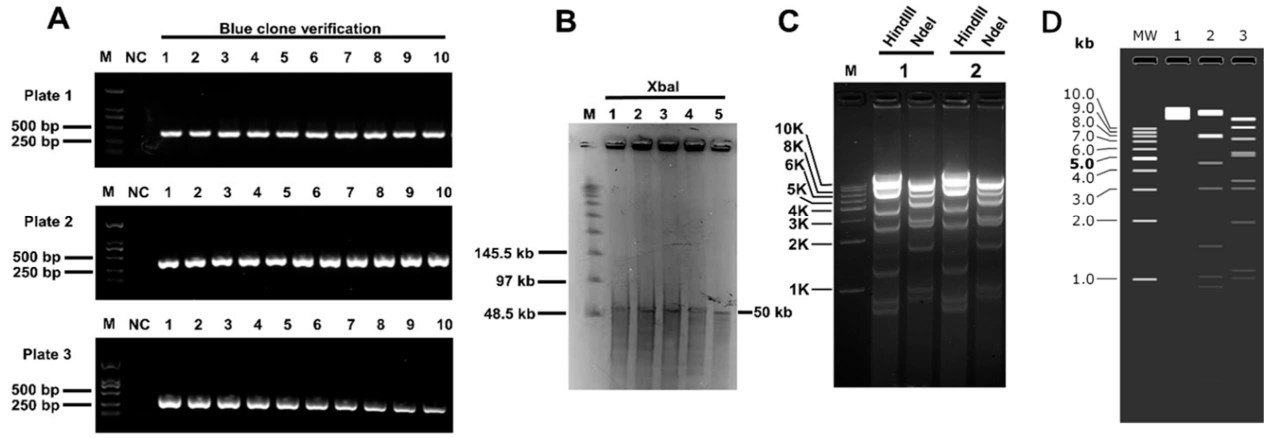

Supplementary Figure S3 The plasmid with 50 kb DNA fragment restriction verification. (A)Colony PCR verification, 10 transformants were randomly screened in three independent replicate cloning experiments for PCR verification. NC: negative control. (B) PFGE analysis for *E. coli* 50 kb after *Xba*I digestion; (C) *E. coli* 50 kb plasmid digestion verification results. (D) Simulate agarose gel. MW: 1 kb DNA Ladder; 1: *E. coli* 50 kb plasmid; 2: *Hind*III; 3: *Nde*I.

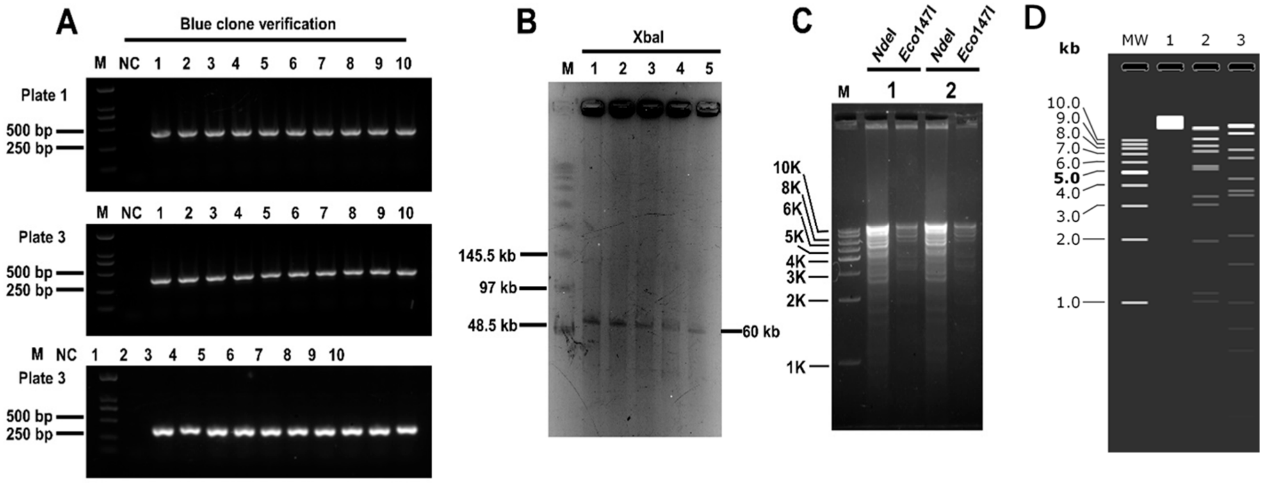

Supplementary Figure S4 The plasmid with 60 kb DNA fragment restriction verification. (A)Colony PCR verification, 10 transformants were randomly screened in three independent replicate cloning experiments for PCR verification. NC: negative control. (B) PFGE analysis for *E. coli* 60 kb after *Xba*I digestion; (C) *E. coli* 60 kb plasmid digestion verification results. (D) Simulate agarose gel. MW: 1 kb DNA Ladder; 1: *E. coli* 60 kb plasmid; 2: *Nde*I; 3: *Eco147*I.

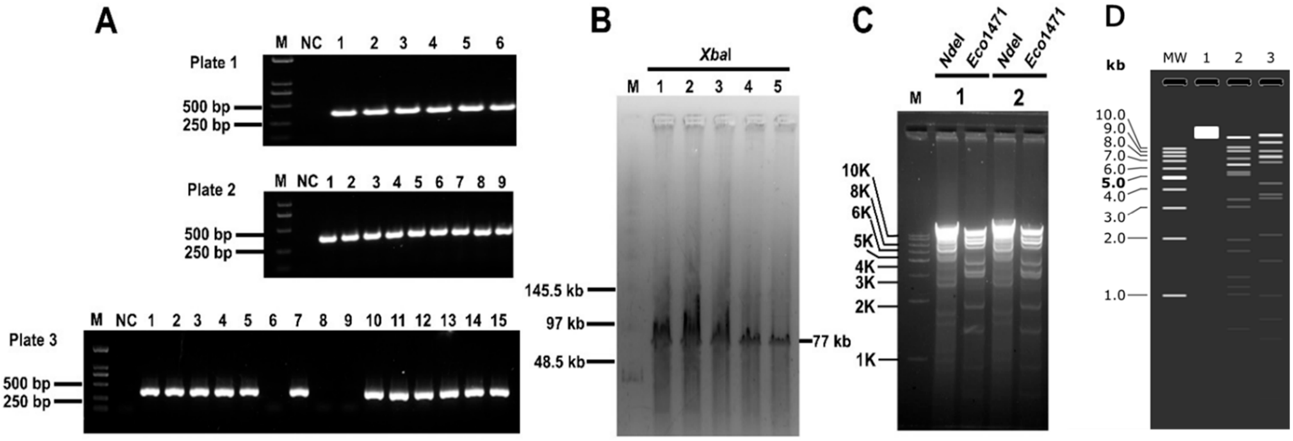

Supplementary Figure S5 The plasmid with 77 kb DNA fragment restriction verification. (A)Colony PCR verification, 10 transformants were randomly screened in three independent replicate cloning experiments for PCR verification. NC: negative control. (B) PFGE analysis for *E. coli* 77 kb after *Xba*I digestion; (C) *E. coli* 77 kb plasmid digestion verification results. (D) Simulate agarose gel. MW: 1 kb DNA Ladder; 1: *E. coli* 60 kb plasmid; 2: *Nde*I; 3: *Eco147*I.
